# Minimally invasive K-wire fixation of displaced intraarticular calcaneal fractures through a minimal sinus tarsi approach

**DOI:** 10.1186/s10195-022-00680-5

**Published:** 2023-01-21

**Authors:** Ayman Youssef Eltabbaa, Mahmoud Abdelmonem El-Rosasy, Mohammed Roshdy El-Tabbakh, Mostafa Naguib Elfakhrany

**Affiliations:** Orthopedic and Traumatology Department, Faculty of Medicine, Tanta University Hospital, Tanta, Egypt

**Keywords:** Intra-articular calcaneal fractures, Minimally invasive fixation, Sinus tarsi approach

## Abstract

**Background:**

Displaced intra-articular calcaneal fractures are challenging injuries, and there is debate regarding the best method of treatment.

**Patients and methods:**

Between January 2018 and January 2021, a prospective study was conducted on 46 patients with 56 displaced intraarticular calcaneal fractures that were treated with minimally invasive fixation using Kirschner wires (KWs) through the sinus tarsi approach.

**Results:**

The mean follow-up period was 22.36 months. The American Orthopaedic Foot and Ankle Society (AOFAS) score was adopted as a method of clinical evaluation; the mean AOFAS score was 78.4. All cases showed radiographic evidence of adequate healing, with no collapse till the final follow-up. Complications included persistent pain, subtalar arthritis, deep infection and superficial pin site infection.

**Conclusion:**

The use of the sinus tarsi approach and percutaneous KWs represents a minimally invasive approach which expands the indications of surgery for displaced intra-articular calcaneal fractures with fewer treatment-related complications.

*Level of evidence* (4) case series.

*Trial registration* This study has been approved by the ethical research committee of the Faculty of Medicine, Tanta University, under the code: 35901/10/22.

## Introduction

Calcaneal fracture is the most common tarsal fracture and is commonly caused by a fall from height [10]. There is no consensus regarding the best treatment strategy for displaced intra-articular calcaneal fractures—operative or conservative treatment, or even the best surgical intervention [24]. However, there has recently been wide agreement that these injuries should be treated surgically to restore the articular surface and the calcaneal morphology, height and width in order to avoid long-term sequelae, especially posttraumatic arthritis, chronic pain and a deformed hind foot [8, 16]. Those long-term problems cause a high economic burden and long-term suffering [2]. Open reduction and internal fixation using the formal extensile lateral approach offers wide exposure with the capacity for direct reduction of the articular surface [18]. Nevertheless, many studies have reported a high morbidity associated with this approach; soft tissue compromise and prominent metal were reported in up to 25% of cases in some studies, especially in smokers and diabetics [3, 4, 12].

On the other hand, closed reduction and percutaneous screw fixation avoids soft tissue problems, although it carries the risk of inappropriate reduction of the articular surface, as intraoperative images could be deceiving [1]. Moreover, rafting screws should be inserted through the subchondral bone and parallel to the articular surface of the posterior subtalar facet to provide true rafting. So, the entry should be from just posterior to the posterior articular facet, which is not practical, and the posterior tuberosity screws do not provide not true rafting [9]. We hypothesized that the ideal treatment method should allow direct visualization and reduction of the articular surface alongside stable fixation while minimizing soft tissue injury.

This study aimed at assessing the clinical and radiological results of minimally invasive fixation of displaced intra-articular calcaneal fractures by KWs through a limited sinus tarsi approach.

### Patients and methods

Between January 2018 and January 2021, a prospective study was conducted on 46 consecutive patients with 56 displaced intra-articular calcaneal fractures. Minimally invasive fixation by Kirschner wires (KWs) through a limited sinus tarsi approach was used in all cases.

Patients included in this study had a closed displaced intra-articular calcaneal fracture, and the injury occurred no more than 2 weeks earlier. Exclusion criteria included patients with evidence of peripheral neuropathy, peripheral vascular insufficiency, chronic debilitating illness and a preexisting ankle or foot injury. Moreover, patients younger than 16 years or those who presented later than 2 weeks were excluded.

**Fig. 1:**
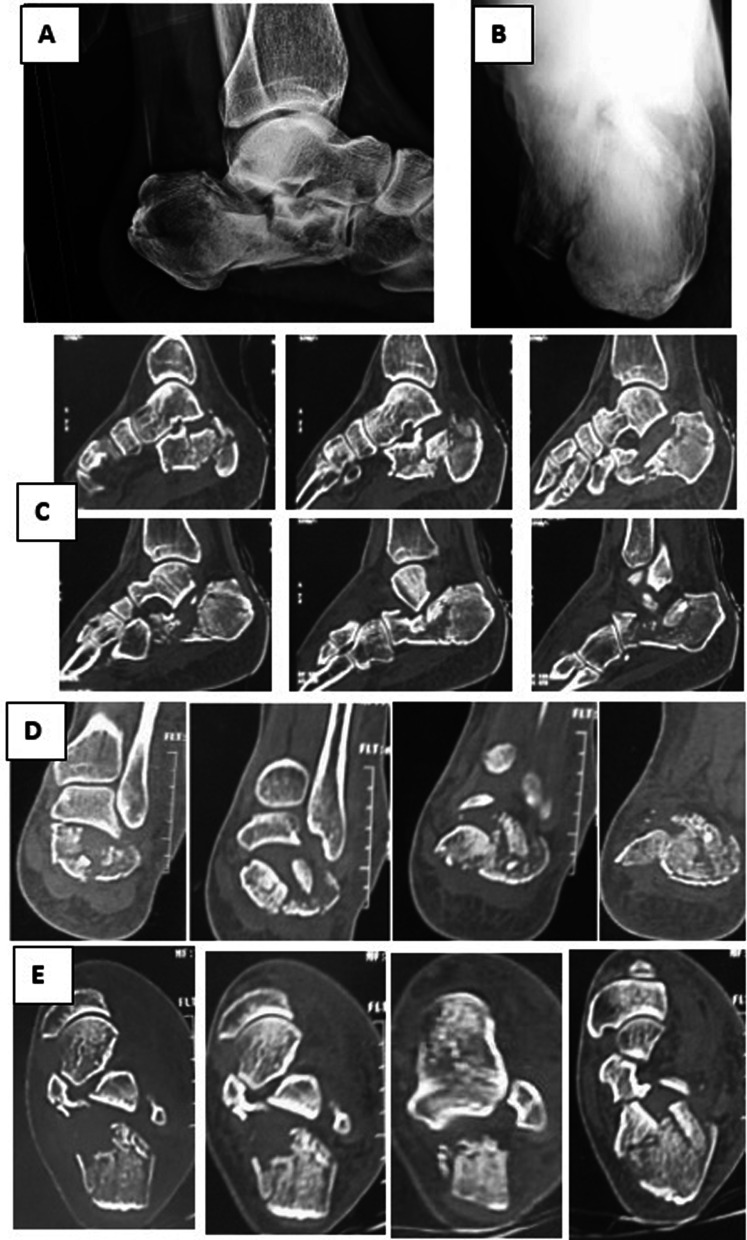
Preoperative radiographs of a displaced intra-articular calcaneal fracture: **A** lateral plain ankle X-ray showing the depressed articular surface of the calcaneus; **B** axial view of the calcaneus; **C**, **D**, **E** sagittal, coronal and axial CT cuts, respectively

All patients had a clinical and radiological evaluation, including X-rays with standard lateral and Harris axial views of the calcaneum in addition to a CT scan with 3D reconstruction (Fig. 1).

Fractures were then classified according to the Sanders classification of intra-articular calcaneus fractures [19]. There were 6 patients (10.7%) with type 2A, 12 patients (21.4%) with type 2B, 6 patients (10.7%) with type 2C, 6 patients (10.7%) with type 3AB, 9 patients (16.1) with type 3 AC, 5 patients (8.9%) with type 3BC and 12 patients (21.4%) with type 4 fractures. There were 40 men and 6 women. Bilateral calcaneal fractures were encountered in 10 patients; 18 fractures were on the right side only and 18 were on the left side only. The mean age was 43.25 ± 4.26 years (ranging from 22 to 64 years). All patients had been counseled about the procedure and a written consent was taken before surgery. Table 1 presents the demographic data on the cases studied.

**Table 1:** Patients’ demographics

Characteristic	Value
Mean age	43.25 ± 4.26 years
Gender	40 men 6 women
Side	36 unilateral 10 bilateral
Mechanism of injury	Fall from height in 40 cases Motor vehicle accident in 6 cases
Smoking	20 smokers 26 nonsmokers
Associated comorbidities	3 uncontrolled diabetes 2 HCV 2 hypertensive
Mean time from injury to surgery	9.6 days
Sanders type	Type 2A: 6 Type 2B: 12 Type 2C: 6 Type 3AB: 6 Type 3AC: 9 Type 3BC: 5 Type 4: 12
Mean follow-up period	22.36 months

*N *= 56 feet in 46 patients

### Surgical technique

A preoperative antibiotic was given at the induction of the anesthesia. The supine position was used, with a bump under the ipsilateral hip to rotate the leg internally on a translucent table so that fluoroscopy could be used intraoperatively. After exsanguination, a pneumatic tourniquet was inflated to 350 mmHg. The sinus tarsi approach [22] was used to expose the subtalar joint. A 3-cm skin incision was made along the sinus tarsi and parallel to the peroneal tendons, which were then retracted plantarward, protecting the sural nerve. Afterwards, the articular surface was clearly visible, and the depressed articular fragment or fragments could be manipulated, dis-impacted and elevated using an elevator [22].

**Fig. 2:**
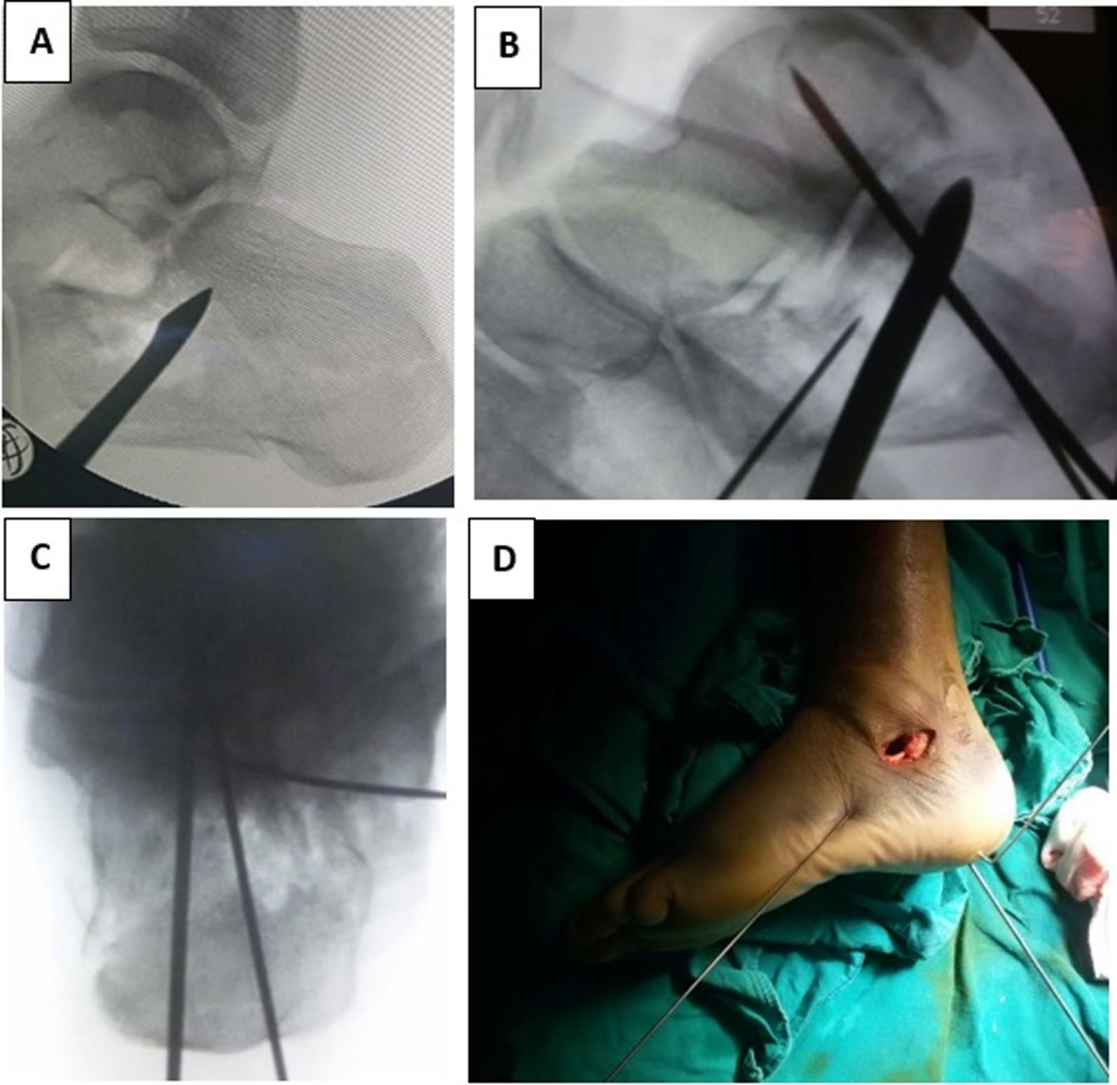
Intraoperative images. **A** Image clarifying the elevation of the depressed articular fragment using a bone elevator. **B**, **C** Lateral and axial fluoroscopic images, respectively, for evaluating the reduction. **D** Clinical image showing the minimally invasive sinus tarsi approach

The reduction of the articular surface was evaluated by using the articular surface of the talus as a template for the reduction, in addition to intraoperative fluoroscopy. Then the reduction was stabilized using one or more trans-articular KWs from the calcaneum to the talus. The reduction and KW positions were then evaluated using lateral, Harris axial and broaden views [23] (Fig. 2).

At the end of the procedure, the tourniquet was released with appropriate hemostasis. In cases where a bone void was found, it was packed with a gel-foam roll. The wound was closed in layers. The KWs were cut approximately 1 cm out from the skin and dressed with protective gauze dressings, and the leg was held in a below-knee back slab. Sutures were removed after 2 weeks, and a below-knee cast was applied for a further 4 weeks. The KWs and cast were removed after 6 weeks from surgery; then range of motion and toe-touch weight bearing was allowed till the end of the third month postoperatively.

### Evaluation of the outcome

**Fig. 3:**
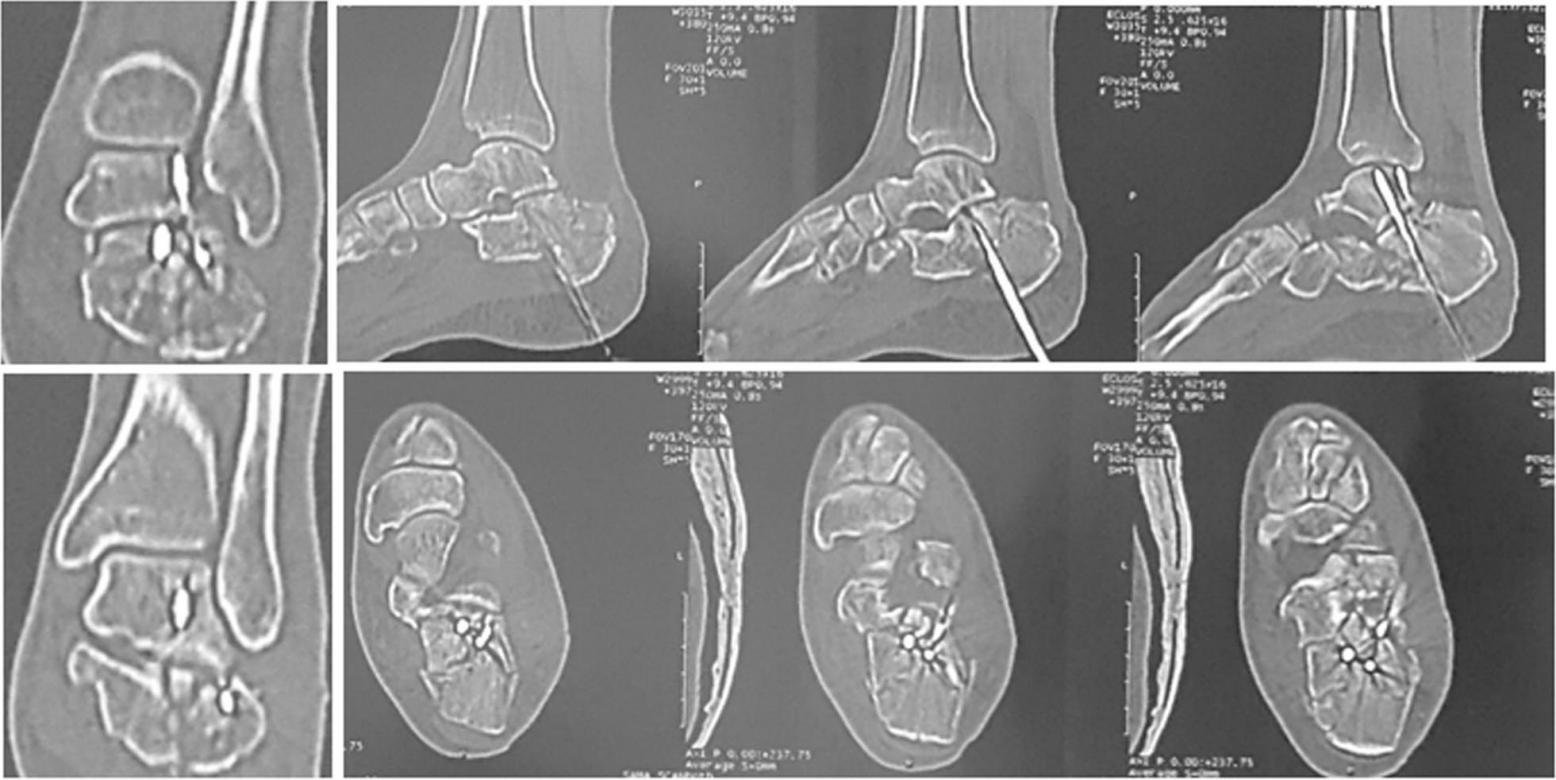
Postoperative CT for evaluating the articular surface reduction

All patients underwent clinical and radiological reviews at 6, 12 and 24 weeks, and at 1 year. Radiographic evaluation included lateral and Harris axial views for assessing the calcaneal morphology, height and width. In addition, the pre-operative and post-operative calcaneal angles were compared [24]. Subtalar arthritis was evaluated in CT scans taken 6 months postoperatively. Clinical evaluation was based upon the American Orthopaedic Foot and Ankle Society ankle–hindfoot score (AOFAS ankle–hindfoot score) [13]. This score consists of 100 points: 40 for pain, 50 for function and 10 points for alignment (Fig. 3).

Statistical analysis was done using the Statistical Package for the Social Sciences (SPSS 17.0). Quantitative data were expressed as mean ± standard deviation (± SD). The chi-square (*X*^2^) test was used to compare proportions between qualitative parameters. A *p* value of < 0.05 was considered significant. Outcome assessors participated in the surgical intervention and were part of the surgical team.

## Results

**Fig. 4:**
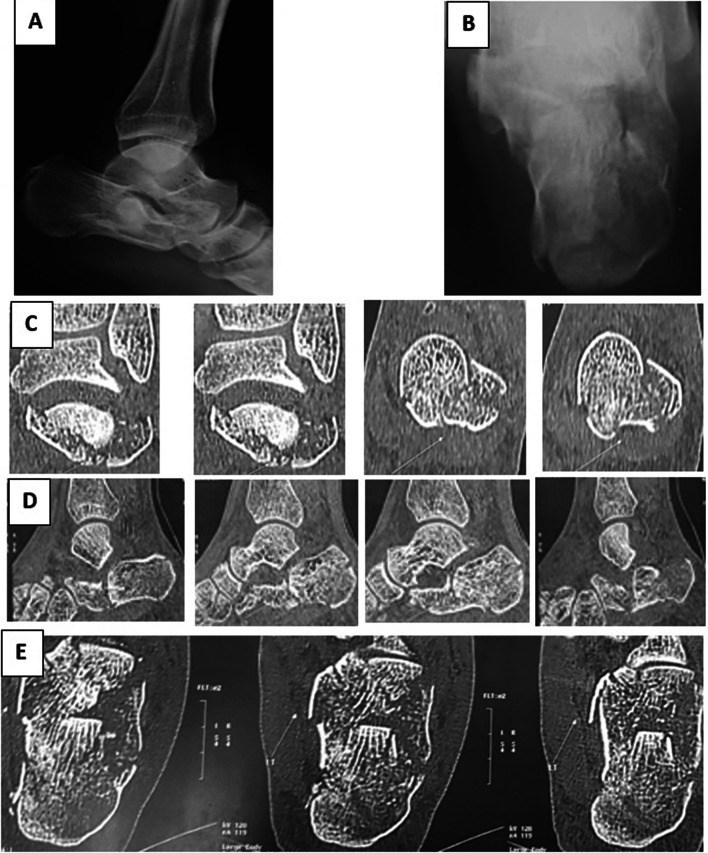
Complete case presentation. **A**, **B** Lateral and axial plain preoperative radiographs, respectively. **C**, **D**, **E** Coronal, sagittal and axial preoperative CT cuts

**Fig. 5:**
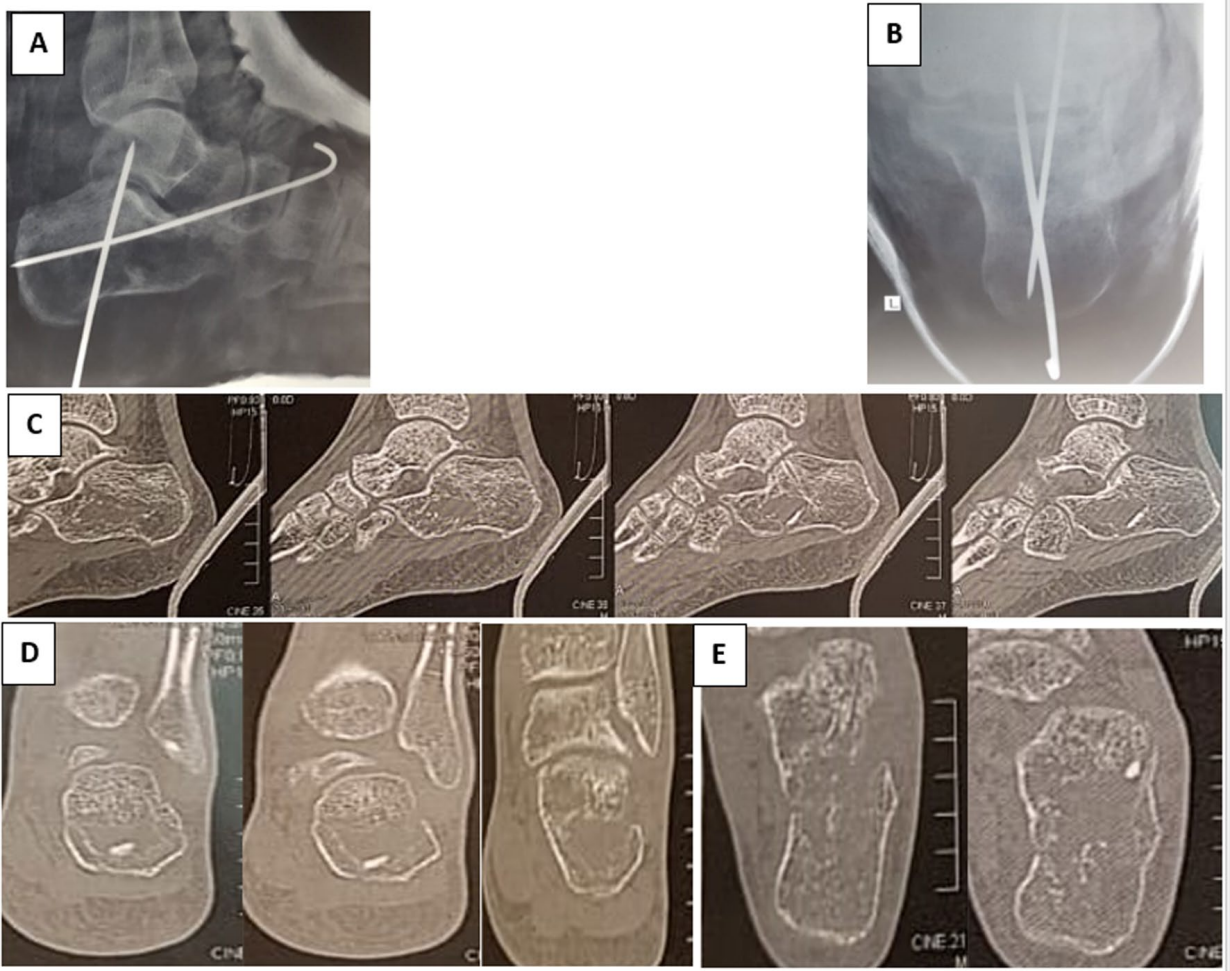
**A**, **B** Immediate postoperative lateral and axial plain X-rays, respectively. **C**, **D**, **E** 6 months postoperative CT

The current study included 56 displaced intra-articular calcaneal fractures. All patients completed a minimum of 12 months follow-up postoperatively. The mean follow-up period was 22.36 months (ranging from 12 to 48 months); see Figs. 4 and 5.

Fall from height was the mechanism of injury in 40 patients (87%), while 6 patients (13%) had calcaneal fractures after a motor vehicle accident. The mean time from trauma to surgery was 9.6 days. Clinical evaluation was based on the AOFAS score. The mean AOFAS score was 78.4 (ranging from 55 to 95). The operative time ranged from 30 to 45 min.

**Fig. 6:**
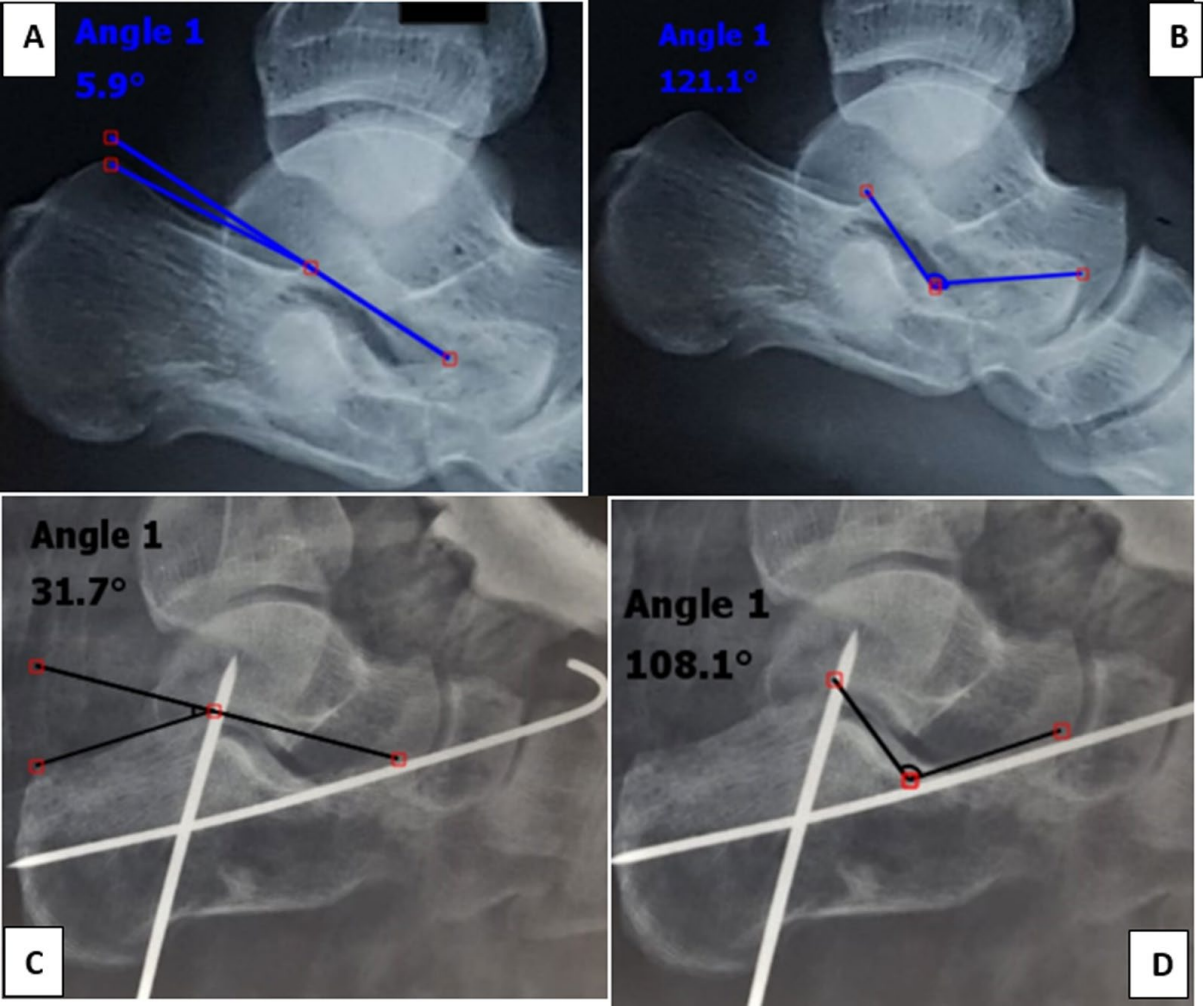
Comparison between the preoperative and postoperative calcaneal angles. **A** Nearly flat preoperative Bohler’s angle. **B** Increased preoperative angle of Gissane. ** C**, **D** Restoration of Bohler’s angle and the angle of Gissane, respectively

As regard to the radiographic evaluation, parallelism between the articular surface of the talus and the posterior calcaneal facet was used as an indication of articular congruity. Moreover, Bohler’s angle and the angle of Gissane were measured. The mean preoperative Bohler’s angle and angle of Gissane were 12.4° and 127.9°, respectively, and the mean postoperative Bohler’s angle and angle of Gissane were 26.3° and 107.3°, respectively. All patients healed within 4 months; no patients had a loss of reduction after wire removal (Fig. 6).

At the end of the study, nine patients (16%) had persistent pain. Subtalar injection relieved the pain successfully in four cases; however, subtalar fusion was needed in five cases (8.9%) due to persistent pain after injection. Infection was encountered in eight patients (14%): two cases of deep infection and six cases of superficial pin site infection. The deep infection was severe in one patient, which necessitated the removal of the wires prematurely, with fair end results. The other patient developed a deep infection a few days after the removal of the wires, as he was a farmer and he worked in his farm a few days after the removal of the pins; complete healing was achieved following surgical debridement. Superficial pin site infection was controlled with oral antibiotics. One patient (1.7%) reported numbness over the lateral aspect of the foot. The results are summarized in Table 2.

**Table 2:** Results

Characteristic	Value
Mean preoperative Bohler’s angle	12.4°
Mean postoperative Bohler’s angle	26.3°
Mean preoperative angle of Gissane	127.9°
Mean postoperative angle of Gissane	107.3°
Mean AOFAS score	78.4 (55–95)
Complications	Persistent pain: 9 cases (16%)
	Subtalar fusion: 5 cases (8.9%)
	Superficial pin site infection: 6 cases (10.7%)
	Deep infection: 2 cases (3.5%)
	Neuralgia: 1 case (1.7%)
Operative time	30–45 min

*N* = 56 feet in 46 patients

## Discussion

There is no consensus regarding the best treatment method for displaced intra-articular calcaneal fractures—operative or conservative—nor the best surgical method. However, it is now widely agreed that the consequences of inadequately treated intra-articular calcaneal fractures can be disabling, including common persistent pain, a distorted hind foot morphology and a delayed return to work [5, 17, 20].

The dilemma of balancing soft tissue compromise with the need for anatomic reduction of the articular surface under direct vision is the whole problem. Several approaches and fixation methods have been described: lateral, medial, double-incision medial and lateral, sinus tarsi and extensile lateral approaches. Fixation modalities have included screws, locked anatomical plates and a specific nail, and some studies have reported using only Kirschner wires [1, 11, 20].

The extensile lateral approach has gained popularity in the last few decades; nevertheless, it has gained a bad reputation owing to wound complications at rates that reached up to 25% in some studies. Wound complications included deep infection, osteomyelitis, wound dehiscence and even amputation. Similar problems have been reported in studies that used double incision, but with a decreased (but still high) rate of wound compromise: 6%. Thus, trends towards minimally invasive approaches have gained some preference recently [7].

In a systemic review by Dingemans et al., it was revealed that different methods of fixation did not differ greatly, and that all methods used have similar rigidities and stabilities. Moreover, they stated that anatomical locked plates that need an extensile approach and maximum soft tissue compromise have no advantage over the other methods described [12].

Although there is no consensus regarding the best fixation method for comminuted intra-articular calcaneal fractures, we have favored using only KWs, owing to some factors. Rigid fixation using screws to compress the articular surface has always been sought. Unfortunately, this is not always achievable in such complex and challenging fractures.

Most of our cases were high-energy traumas caused by a fall from height (40/46), with the majority being Sanders type 3 or 4 (32/56). Comminuted calcaneal fractures were commonly associated with rupture of the lateral cortical wall, alongside a lack of structural support of the subchondral cancellous bone, which was usually impacted, rendering a big cavity underneath a small articular fragment. We had concerns that using screws in such comminuted unsupported small fragments would not only lead to weak and doubtful purchase but might also split those fragments into more comminuted ones.

On the other hand, using KWs gave us the advantage of supporting each individual fragment, which might not be possible with screws. Using KWs allowed us to transfix those small articular fragments to the talus, giving more support. In addition to a shorter operative time, this avoids the problem of prominent metal, which is not uncommon after calcaneal fixation, and allows easy removal as part of the outpatient follow-up.

The ideal treatment method seems to have minimal soft tissue compromise, restores the general hind foot morphology, reduces the calcaneal articular surface under direct vision, and has stable fixation. In our study, we used a limited sinus tarsi approach to reduce the articular surface of the posterior facet under direct vision. Even articular fragments of the medial part of the posterior facet were accessed and reduced using the same incision. We used KWs to transfix the articular surface to the talus. This was stable, with no cases of a loss of reduction observed in the follow-up.

The operative time ranged from 30 to 45 min, which was much less than the 120 min needed in the extensile approach [21] and the 60 min needed in a study that used a minimally invasive approach [27].

At the final follow-up, the mean AOFAS score was 78.4, which was comparable to results achieved by open reduction through the extensile lateral approach or double-incision approach, in addition to previous studies using minimally invasive fixation through the sinus tarsi approach [11, 15, 26]. It should also be taken into consideration that we treated all Sanders groups, including high-risk diabetic and smoker patients, unlike other studies that treated specific Sanders groups and excluded high-risk patients [14]. Moreover, it was found that there is no statistically significant relationship between the Sanders classification and the overall AOFAS score. The lowest AOFAS scores were attributed to some factors: smoking and delay before surgery. Bilateral cases also scored less than unilateral cases, but it should be noted that bilateral fractures are caused by higher-energy trauma, so they are commonly accompanied by spinal or other fractures; also, the lack of a healthy contralateral calcaneus can augment the functional loss in comparison to unilateral cases.

We adopted the AOFAS score in addition to radiographic evaluation as the study then included different types of clinical assessment. Depending solely on radiographic evaluations of Bohler’s angle and the angle of Gissane did not seem to be a fair evaluation [7]. We emphasized that restoration of the general hind foot morphology is as important as the reduction of the calcaneal articular surface and angles [6]. Clearly, this can explain the fact that many patients have radiographic evidence of subtalar degenerative joint disease yet are clinically asymptomatic. This can explain our results: at the final follow-up, 19 patients had radiographic evidence of degenerative joint disease, but only 9 patients were symptomatic.

We had eight cases of infection: a rate of 14%, which is still lower than in studies which used the extensile lateral approach and the double-incision approach, where the infection rates reached up to 40% [7].

Our decision not to use a bone graft is supported by many previous studies. A previous cadaveric study of 14 cadaveric calcanei revealed that there was physiological cavitation under the thalamic portion, which corresponded to the neutral triangle, in 40% of the specimens. In 60% of the specimens, there were sparse bone trabeculae [24, 25].

Unlike other studies that used the same technique, we supported the evaluation of our results by performing CT at the 1-year follow-up. We also treated all fracture types and all patients using the same technique. Nonetheless, this study has some limitations, as we lacked a control group, and this is a technically demanding surgery with a high learning curve.

Considering our results, we believe that the limited sinus tarsi approach for open reduction and percutaneous KW fixation expands the indications of surgery for displaced intra-articular calcaneal fractures with fewer treatment-related complications. This approach allows reduction under direct vision while maximally preserving the soft tissue envelope.

Clinical and radiological assessment seems to be promising and can be used for future randomized controlled trials or prospective cohort studies that focus on the same or similar conditions.

## Data Availability

The datasets used and/or analyzed during the current study are available from the corresponding author on reasonable request.

## Abbreviations

KWs: Kirschner wires
AOFAS: American Orthopaedic Foot and Ankle Society
SPSS: Statistical Package for the Social Sciences
CT: Computed tomography
3D: Three-dimensional
SD: Standard deviation
